# solveME: fast and reliable solution of nonlinear ME models

**DOI:** 10.1186/s12859-016-1240-1

**Published:** 2016-09-22

**Authors:** Laurence Yang, Ding Ma, Ali Ebrahim, Colton J. Lloyd, Michael A. Saunders, Bernhard O. Palsson

**Affiliations:** 1 Department of Bioengineering, University of California at San Diego, La Jolla, 92093 CA USA; 2 Department of Management Science and Engineering, Stanford University, Stanford, 94305 CA USA; 3 Novo Nordisk Foundation Center for Biosustainability, Technical University of Denmark, Kemitorvet, Building 220, Kongens Lyngby, DK-2800 Denmark

**Keywords:** Nonlinear optimization, Constraint-based modeling, Metabolism, Proteome, Quasiconvex

## Abstract

**Background:**

Genome-scale models of metabolism and macromolecular expression (ME) significantly expand the scope and predictive capabilities of constraint-based modeling. ME models present considerable computational challenges: they are much (>30 times) larger than corresponding metabolic reconstructions (M models), are multiscale, and growth maximization is a nonlinear programming (NLP) problem, mainly due to macromolecule dilution constraints.

**Results:**

Here, we address these computational challenges. We develop a fast and numerically reliable solution method for growth maximization in ME models using a quad-precision NLP solver (Quad MINOS). Our method was up to 45 % faster than binary search for six significant digits in growth rate. We also develop a fast, quad-precision flux variability analysis that is accelerated (up to 60× speedup) via solver warm-starts. Finally, we employ the tools developed to investigate growth-coupled succinate overproduction, accounting for proteome constraints.

**Conclusions:**

Just as genome-scale metabolic reconstructions have become an invaluable tool for computational and systems biologists, we anticipate that these fast and numerically reliable ME solution methods will accelerate the wide-spread adoption of ME models for researchers in these fields.

**Electronic supplementary material:**

The online version of this article (doi:10.1186/s12859-016-1240-1) contains supplementary material, which is available to authorized users.

## Background

Constraint-based reconstruction and analysis (COBRA) methods enable systems level computation of cellular functions using genome-scale biochemical reaction networks, including metabolism [1]. Metabolic network reconstructions are used to compute reaction fluxes at pseudo steady-state by solving the linear program,

$$\begin{array}{*{20}l} \max_{v} \quad & c^{T} v \\ \mathrm{s.t.} \quad & Sv = 0, \\ & v^{L} \leq v \leq v^{U}, \end{array} $$

where *v* is the vector of fluxes, *v*^*L*^ and *v*^*U*^ are lower and upper flux bounds, *S* is the stoichiometric matrix, and *c* is the vector of objective coefficients (e.g., to maximize growth rate). We refer to [2] for details on COBRA for metabolic networks.

Recently, Lerman et al. [3] developed the first integrated genome-scale reconstruction of Metabolism and macromolecular Expression (ME) for the microorganism *Thermotoga maritima*. This ME model described the transcription and translation machinery associated with 651 genes and the metabolic network catalyzed by the enzymes synthesized in the model. Thereafter, Thiele et al. [4] developed the first ME model for *Escherichia coli*, which was followed by additional ME models for *E. coli* by [5] and [6]. The latest ME models for *E. coli* account for 80 % of the proteome by mass [5], enable computation of proteome allocation shifts between conditions [7], and predict the macromolecular composition of the cell [8].

While ME models are a significant advancement for COBRA modeling, they pose challenging mathematical optimization problems because of their size and multiscale nature. In particular, the vastly different magnitudes of metabolic and expression machinery fluxes lead to ill-conditioned problems that cause difficulties for standard optimization solvers: feasible problems can be reported as infeasible, or solutions may contain numerical errors. Methods have been developed to enable off-the-shelf linear programming (LP) solvers to be used for solving ME models [9]. Additionally, quad-precision LP solvers have been recently applied to ME models [10]. They present an attractive balance between reliability and speed for practical solution of ME models.

The growth rate maximization problem for ME models is actually a nonlinear program (NLP) but it becomes an LP when the growth rate *μ* is fixed. So far, the NLP has been solved using binary search on *μ* [5], with high-precision LP solvers [11] solving a sequence of LP subproblems. Thus, a remaining question has been whether NLP solvers could solve the NLP more expediently. The solution of NLPs in quad-precision has only recently become possible due to the development of quad-precision MINOS (Quad MINOS) [10]. However, the NLP capabilities of Quad MINOS have not yet been tested for multiscale ME models (only the LP capabilities have been reported in [10]). We thus report the first study for solving the nonlinear growth maximization problem for ME models using NLP methods in quad precision. With a suitable initial estimate of *μ*, the quad-precision NLP approach proves to be both fast and numerically reliable.

## Results

We developed several solution methods for solving linear and nonlinear programs involving ME models. These methods are listed in Table 1, and are discussed in detail in subsequent sections.

**Table 1:** Glossary of solution methods

Method	Description
bisectME	Maximizes growth rate *μ* via binary or golden section search [15]. Uses Quad MINOS to solve LP subproblems in quad precision [10]. Every LP after the first is warm-started using the previous solution.
solveME	Combined solution procedure for the nonlinear growth-rate maximization problem Eq. (1). Uses bisectME to find a feasible estimate *μ* ^0^, which is used to compute *μ* (and all fluxes) to high precision using the Quad MINOS NLP solver.
varyME	Flux variability analysis [16] in quad precision with solver warm-starts [17].

### NLP formulation and its global optimum

The growth rate maximization problem in a ME model is typically a nonlinear program because macromolecule dilution is modeled using the product of growth rate with a continuous reaction flux [5]. Specifically, the ME problem of maximizing growth rate is formulated as follows:

$${} \begin{aligned} \max_{\mu,\,v} \ & \mu\\ \mathrm{s.t.} \ & Sv = 0, \quad v^{L} \leq v \leq v^{U}\\ &\!\! v_{\text{dilution},\text{ribo}} \geq\! \sum_{i \in \mathit{Peptide}}\!\left(\!\frac{l_{p,i}}{c_{\text{ribo}}\kappa_{\tau}}\left(\mu+r_{0}\kappa_{\tau}\right) \cdot v_{\text{translation},i} \! \right)\\ &\!\! v_{\text{dilution},\text{RNAP}} \geq\!\! \sum_{i \in \mathit{TU}}\!\!\left(\! \frac{l_{\text{TU},i}}{3 c_{\text{ribo}}\kappa_{\tau}}\!\left(\mu+r_{0}\kappa_{\tau}\!\right) \cdot v_{\text{transcription},i}\! \right)\\ &\!\! v_{\text{dilution},j} \geq \frac{1}{\kappa_{\tau} c_{\text{tRNA},j}} \left(\mu + \kappa_{\tau} r_{0}\right) v_{\text{charging},j},\quad\!\! \forall j \in \mathit{tRNA}\\ &\!\! v_{\text{dilution},j} \geq \mu \sum_{i} \left(\frac{1}{k^{\text{eff}}_{ij}} v_{\text{usage},i} \right), \quad \forall j \in \mathit{Enzyme}\\ &\!\! \textrm{additional \(\mu\)-dependent constraints}, \end{aligned} $$

where *μ* is the growth rate and *v* is the vector of all fluxes; *l*_*p*,*i*_ is the length of peptide *i* and *l*_TU,*i*_ is the length of transcription unit *i*; *c*_ribo_, *κ*_*τ*_, *r*_0_, $k^{\text {eff}}_{ij}$, *c*_mRNA,*j*_, *c*_tRNA,*j*_ are constants; and *Peptide*, *TU*, *tRNA*, *Enzyme* are index sets of *v* representing peptides, transcription units, tRNA, and enzymes, respectively.

Collecting all linear and nonlinear constraints, we formulate the nonlinear ME problem in the following generalized form (Eq. 1):

1 $$\begin{array}{*{20}l} \max_{\mu,\,v} \ & \mu \\ \mathrm{s.t.} \ & \mu A v + B v = 0\\ & \quad S v = 0, \quad v^{L} \leq v \leq v^{U}, \end{array} $$

where the constraints *μAv*+*Bv*=0 are quasiconvex (quasilinear, in fact—see “Methods”).

An optimal solution *μ*^∗^ can be found using binary search (bisection) as in [5]. Dattorro [12] proves that *μ*^∗^ is a global optimum. With *a*<*μ*^∗^<*b* and initial estimate *μ*^0^∈[*a*,*b*], bisection converges to specified accuracy *ε* in log2((*b*−*a*)/*ε*) iterations. We implemented bisection as Algorithm bisectME using the simplex method in quad precision (Quad MINOS [10]).

Faster convergence to the global optimum is possible with an appropriate NLP solver. Specifically, Quad MINOS is a quad-precision implementation of MINOS [13]. For problem Eq. (1), MINOS solves a sequence of linearly constrained subproblems defined by linearizing the constraints at a sequence of approximate solutions {*μ*^*k*^, *k*=0,1,2,… }. (The objective function for each subproblem is an augmented Lagrangian.) If MINOS converges, it will be to a global optimum *μ*^∗^. Furthermore, the subproblems converge quadratically when *μ*^*k*^ is close enough to *μ*^∗^ (Robinson [14]).

**Fig. 1:**
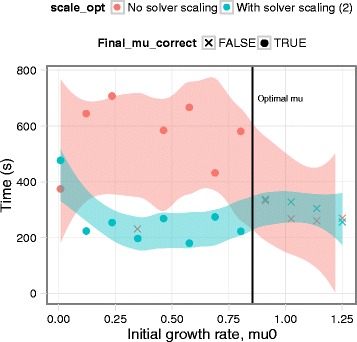
Effect of initial solution and scaling. Shading represents 90 % confidence intervals for a loess fit of the data points computed using ggplot2 in R. When scaling was used, this solver option was set to a value of 2 (series of alternating row and column scaling with additional scaling)

We tested the effect of *μ*^0^ and the MINOS scaling option on the computation time and final solution. Empirical tests showed that *μ*^0^=0.0 should be avoided, as should *μ*^0^>*μ*^∗^ (Fig. 1).

The latter is understandable in terms of the algorithm used by MINOS to handle nonlinear constraints [13]. By definition, the constraints are infeasible when *μ*>*μ*^∗^. Hence, if the constraints are linearized at *μ*^0^>*μ*^∗^, they will be infeasible. The MINOS algorithm is not well-defined in this circumstance. As expected, the solution time was generally shorter when *μ*^0^ was closer to *μ*^∗^.

### solveME: Combined solution procedure for growth maximization NLP

Because Quad MINOS converges faster when *μ*^0^ is closer to the optimum, we developed a combined solution procedure that uses a coarse bisection via bisectME to identify *μ*^0^<*μ*^∗^, then provides the corresponding basis to warm-start Quad MINOS on the NLP. This procedure is described in Algorithm 1.



For the bisectME phase, we implemented golden section search (GSS) [15] for improved efficiency (fewer quad-LP evaluations). Specifically, we found that for 3-decimal precision in growth rate, GSS required 81 seconds, while binary search required 98 seconds, representing a 17 % speedup. Whether using golden section or binary, bisectME produces a basis compatible with the NLP solver, enabling warm-start of the NLP. Hence we used GSS to find a low-precision estimate *μ*^0^ for the NLP. The resulting basis and approximate *μ* were used to warm-start Eq. (1) reliably and efficiently at a *μ*^0^<*μ*^∗^.

**Fig. 2:**
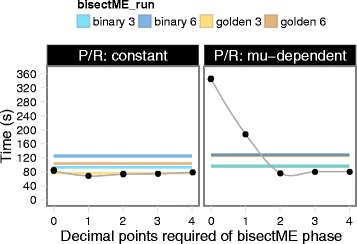
Performance of the combined solveME procedure. Colored bands represent the range of solution times (3 replicate runs) for the binary or golden section search methods with growth rate convergence to 3 or 6 decimal points. Points show solution time for the combined solveME procedure (3 replicate runs each) for varying decimal points required at the bisectME phase. The protein-per-RNA ratio (P/R) in the ME model was either constant (*left*) or growth-rate dependent as in [5] (*right*). Uptake rates were free in both cases, such that proteome limitation eventually constrained uptake rates

The combined solveME procedure took 68 to 85 s (Fig. 2), depending on the accuracy requested in bisectME. Empirically, zero decimals led to the longest solve time, while the fastest solve was achieved when one decimal was requested (two decimals when protein-per-RNA ratio was *μ*-dependent) (Fig. 2). For more decimals, the time spent in bisectME offset the advantages of starting the NLP closer to the optimum.

Compared to the combined solveME, bisectME (with golden section search) took an average of 75 and 103 s for 3 and 6 decimal points respectively, while binary search (not GSS) required an average of 93 and 124 s for 3- and 6-digit precision in growth rate. All runs were performed in quad-precision using Quad MINOS.

In summary, the combined solveME was up to 10 and 34 % faster than golden section search, or 27 and 45 % faster than binary search, for 3- and 6-digit accuracy in growth rate. Another advantage of the combined solveME over bisection methods was that the growth rate was always returned to about 15 digits because of the tolerances set for Quad MINOS; therefore solution time was not a function of final solution precision. To reach the same precision, bisection would require about 50 iterations, or over 400 s. Furthermore, double-precision solvers would have difficulty achieving even 6 or 7 digits.

In particular, commercial double-precision solvers such as CPLEX and Gurobi handle feasibility and optimality tolerances as small as 10^−9^ but are not certain to achieve them. Using quad-precision, we achieved feasibility and optimality tolerances below 10^−15^, and often less than 10^−20^.

### varyME: quad-precision ME variability analysis

In addition to growth rate maximization, we developed quad-precision flux variability analysis (FVA) [16] for ME models, referred to as varyME. As with the fastFVA method [17] for metabolic reconstructions, we decreased the computation time by warm-starting Quad MINOS using the basis from previous LP solutions.

#### Improved solution reliability via quad-precision

Empirical tests demonstrated the importance of quad-precision when FVA is applied to ME models. Specifically, without quad-precision, errors such as maximum flux being less than the minimum flux were observed (Table 2). Another example is the varyME result for translation of b0071 and b0072, encoding for LeuC and LeuD, respectively. LeuC and LeuD are the two subunits for the isopropylmalate isomerase complex. The formation of this complex is the only sink for the two translation products, other than dilution; therefore, FVA should return equal values for the two translation fluxes. Indeed, Quad MINOS correctly determined equivalent FVA solutions for b0071 and b0072, while double-precision runs using CPLEX led to different FVA solutions (Table 2). Additionally, at maximum growth rate, the ME model showed essentially zero variability in the translation rates because of the tendency of ME models to choose only the most efficient proteins. This phenomenon was accurately captured in quad-precision but not in double-precision (Table 2).

**Table 2:** Comparison of FVA in quad- and double-precision at maximum growth rate

		Quad-precision		Double-precision	
Reaction	Protein	vmin (nmol/gDW/h)	vmax (nmol/gDW/h)	vmin (nmol/gDW/h)	vmax (nmol/gDW/h)
translation_b0169	RpsB	30.719225	30.719225	30.715011	30.712581
translation_b0025	RibF	0.210161	0.210161	0.212807	0.211712
translation_b0071	LeuD	0.303634	0.303634	0.303304	0.765585
translation_b0072	LeuC	0.303634	0.303634	0.303304	0.681146

Note that the flux units are in nanomoles/gDW/h

**Fig. 3:**
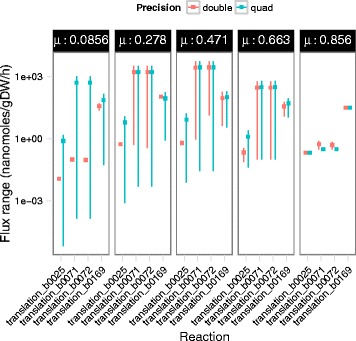
Comparison of FVA in quad- and double-precision. Growth rates were varied from 10 to 100 % of the maximum). Lines span the minimum and maximum fluxes, while points show the mid-point between these ranges. The y-axis is in log-scale

When varyME was performed at growth rates below the maximum, non-zero flux variability was observed (Fig. 3). Again, double-precision ran into numerical difficulties. For example, at lower growth rates, the flux variability of most reactions should be wider, as correctly predicted by Quad MINOS; however, double-precision (CPLEX) runs sporadically predicted narrower ranges at lower growth rates, presumably due to the numerical difficulties associated with the multiscale ME model. Collectively, these results emphasize the importance and practical utility of quad-precision for ME models.

Finally, we tested the speedup of flux variability analysis when each LP was warm-started (as in [17]) using the basis of the previous LP instead of “cold-starting” every LP. We observed 60× and 25× speedup for the *E. coli* and *T. maritima* ME models, respectively (Table 3). This result confirmed the effectiveness of warm-starting LPs as observed by [17] and is the default mode for varyME.

**Table 3:** Running time (seconds) for warm-start versus cold-started FVA in quad-precision using Quad MINOS (two LPs are run for each reaction)

Model	Reactions	Warm-start	Cold-start	Speed-up
*E. coli* ME (reduced)	16126	11.2 h	>670 h	60 ×
*T. maritima* ME [3]	17535	∼78 h	>1940 h	25×

### Case study: Proteome-accounting for growth-coupled biochemical overproduction

ME models have imminent utility for systems metabolic engineering. In particular, biochemical overproduction strain design often involves gene knockouts and modulating the expression of production pathways. These genetic manipulations impact host fitness in part by forcing reallocation of the host proteome away from growth and stress response functions. ME models now allow genome-wide accounting of proteome reallocation for engineered strains.

To demonstrate, we analyzed the overproduction of succinate using growth-coupled designs that had been found using a metabolic reconstruction [18, 19]. In particular, the designs involved succinate dehyrogenase (*sdhCDAB*) knockout, together with overexpression of fumarate reductase (*frdABCD*), isocitrate lyase (*aceA*), or both.

**Fig. 4:**
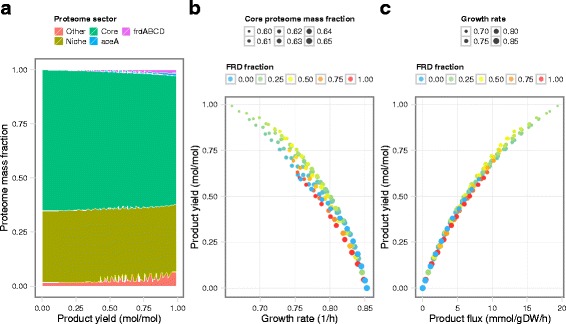
Growth-coupled succinate production performance predicted by solveME. **a** Mass fraction of proteome sectors as a function of product yield. **b** Product yield vs. maximum growth rate. **c** Product yield vs. product flux. Colors in (**b**) and (**c**) are proportional to the FRD fraction, which is the mole fraction of translation flux to FRD vs. total flux to succinate production pathways (FRD and AceA)

To delete *sdhCDAB*, we set zero upper bounds for the translation reactions of the four *sdhCDAB* subunit genes. Overexpression of *frdABCD* and *aceA* was performed in three steps. First, we determined the maximum growth rate with *sdhCDAB* deleted. We then used varyME to determine the feasible flux ranges for the complex formation fluxes of the overexpressed enzymes with growth rate fixed to a small fraction (i.e., 0.05) of the maximum. We then set the lower bound of complex formation fluxes to linearly spaced fractions of the maximum expression flux, from 0.05 to 0.95. Since we were investigating two production pathways, we sampled a grid of 11 by 11 samples for a total of 121 combinations of pathway expression levels. For each expression combination, we solved Eq. (1) for maximum growth rate. Because the strain designs were growth-coupled, we observed varying amounts of succinate production (Fig. 4).

The ME model revealed trends in proteome re-allocation with increasing product yield. Specifically, we traced the relative mass fraction of five proteome sectors, by summing the product of translation rate (mmol/gDW/h) with the protein molecular weight (g/mol) over all proteins in each sector. The core proteome and glucose-niche proteome sector definitions were based on [20]. The core proteome mass fraction, which is critical for cell growth under all conditions, decreased steadily with increasing product yield (Fig. 4a). The niche proteome, which is required for growth on specific environmental niches (in this case, glucose minimal medium), showed more variation with increasing product yield. The two production pathway protein sectors (*frdABCD* and *aceA*) increased with product yield, as expected. Interestingly, the remaining (Other) proteome increased in mass fraction up to four-fold, from 1.6 to 6.7 %, as product yield increased. Therefore, production pathway overexpression entailed a significant proteomic cost.

At lower product yields, the molar ratio of *frdABCD* vs. *aceA* translation rates varied strongly, such that only one pathway was expressed at varying expression levels. However, higher succinate yields were achieved when both pathways were expressed simultaneously at a relatively constant ratio (Fig. 4). This prediction is consistent with experiments showing that simultaneous expression of these two pathways (reductive TCA and glyoxylate shunt) led to higher yields [21]. This ME prediction is also distinct from metabolic reconstructions, which predict the highest yields from utilizing only the most metabolically efficient pathway (i.e., reductive TCA) [19]. The ME model also predicted that product yield experiences diminishing returns with increased product flux, and with decreasing growth rate (Fig. 4b, c). Therefore, the ME model suggests that simultaneous expression of multiple pathways may improve yield over a single metabolically efficient pathway when we account for pathway expression and proteome reallocation requirements.

### Simulation of growth on diverse media and gene deletions

Our solution methods were tested on additional case studies involving large numbers of simulations. First, we performed growth simulations for 1423 single gene knockouts on glucose minimal medium. These essentiality predictions were 89.9 % accurate (73.5 % precision, 50.5 % recall, 96.8 % specificity) (Additional file 2: Table S1), similar to the reference model (iOL1650) accuracy [5]. As with varyME, we tested the speedup of warm-starting the LPs for single gene deletion analysis over cold-starting every LP. We observed 19× and 12× speedup for *E. coli* and *T. maritima* ME models, respectively (Table 4).

**Table 4:** Running time (seconds) for warm-start versus cold-started gene essentiality analysis in quad-precision using Quad MINOS (one LP is run for each reaction at a single growth rate)

Model	Genes	Warm-start	Cold-start	Speed-up
*E. coli* ME (reduced)	1424	3130.8	58967.4	19×
*T. maritima* ME [3]	613^a^	5.26 h	>60 h	12×

^a^Only the 613 genes coding for proteins forming functional complexes were tested for essentiality

**Fig. 5:**
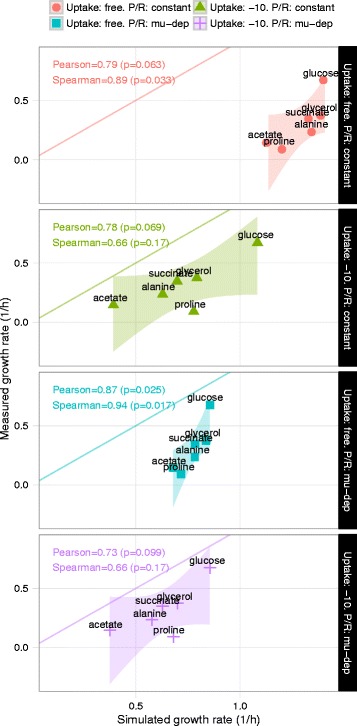
Accuracy of quantitative growth rate predictions. Each panel corresponds to whether a constant or growth rate-dependent P/R ratio was used, and whether uptake rates were left unconstrained (proteome-limited growth) or constrained to −10 mmol/gDW/h. The lines correspond to perfect agreement between measured and simulated growth rates. Colored shadings represent 95 % confidence intervals for a linear model fit of the data points computed using ggplot2 in R

We also predicted relative growth rates across carbon sources accurately. In particular, using a growth rate-dependent protein-per-RNA (P/R) ratio [5] led to the highest accuracy: Spearman rank correlation of 0.94 (*p*=0.017), and Pearson correlation of 0.87 (*p*=0.025) (Fig. 5). Using a constant P/R ratio without hard uptake rate constraints, or artificially constraining uptake rates, led to lower accuracy (Fig. 5). We also observed that the growth rate on L-proline was predicted better when proteome limitation became the active constraint, rather than arbitrary uptake rate constraints. Additionally, biomass yield (grams dry weight per mmol substrate) was predicted accurately across eight carbon sources (Pearson correlation: 0.94, *p*=4.8×10^−4^) (Additional file 1: Figure S1, Additional file 2: Table S2).

**Fig. 6:**
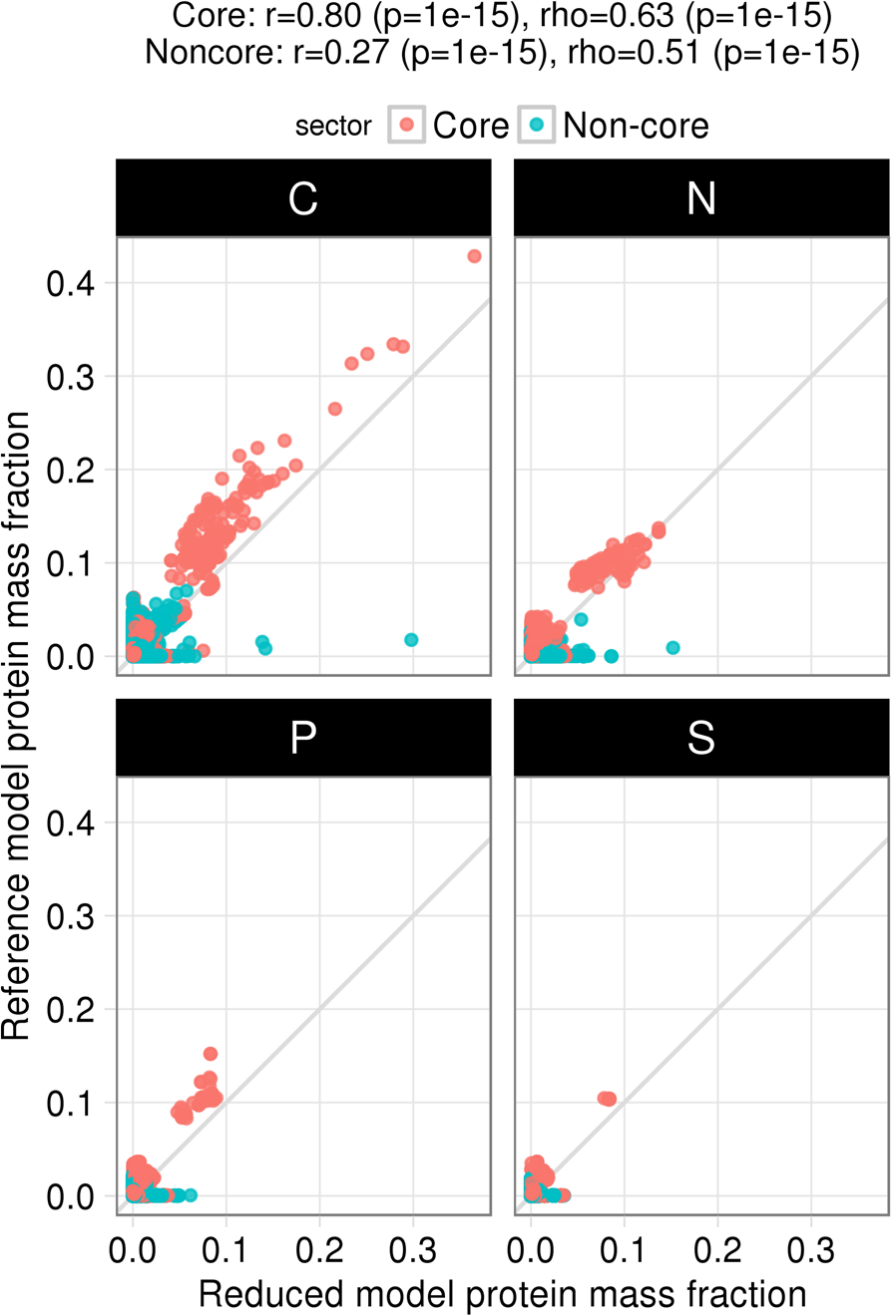
Proteome mass fraction comparision with iOL1650 from [5]. The Pearson (r) and Spearman (rho) correlation coefficients are shown for core and non-core proteome sectors, as defined in [20]. Growth maximization simulations were performed for 333 media conditions, each with a different C, N, P, or S source. Protein mass fractions from the reference model (iOL1650) were obtained from [20]

We also simulated growth on 333 different media, each differing in a carbon, nitrogen, phosphorus, or sulfur source as in [20]. We then compared the predicted emass fractions of the 1423 proteins common between our simulations and similar simulations performed on the iOL1650 ME model in [20]. Overall, we observed Pearson correlation of 0.68 (*p*<10^−15^) and Spearman rank correlation of 0.68 (*p*<10^−15^). When considering only the core proteome [20], we observed higher correlation between the two models: Pearson correlation of 0.80 (*p*<10^−15^) and Spearman rank correlation of 0.63 (*p*<10^−15^) (Fig. 6). These results re-confirmed that ME models (even reduced ones) accurately represent the core proteome [20], which is critical for host cell fitness in natural and engineered contexts.

Finally, throughout these simulations we found that ME fluxes could span 15 orders of magnitude (Table 5) and possibly more depending on the growth condition, emphasizing the need for quad-precision.

**Table 5:** Orders of magnitude of ME reaction fluxes (in mmol/gDW/h). Based on aerobic glucose minimal medium simulation with feasibility and optimality tolerances of 10^−20^. Metabolic processes are categorized by subsystem [37]

Biological process	log10(*v*) min	log10(*v*) max	Median flux	Mean flux
Transcription	–14	–4	2.33E-6	6.46E-6
Complex formation	–14	–4	4.92E-6	1.67E-5
Translation	–14	–4	1.01E-5	2.18E-5
Cell Wall/Membrane/Envelope Metabolism	–7	–7	1.78E-7	1.78E-7
tRNA charging	–7	–1	0.0437	0.0559
Cofactor, Prosthetic Group and Folate Metabolism	–7	0	2.48E-5	0.0915
Amino Acid Metabolism	–6	1	0.159	0.679
Carbohydrate Metabolism	–5	1	2.48	2.74
Energy Production and Conversion	–1	1	5.82	11.5

## Discussion

Genome-scale models of metabolism and macromolecular expression (ME models) [5, 6] have significantly expanded the biological scope and predictive capabilities of constraint-based modeling [8]. While predicting microbial growth using genome-scale metabolic reconstructions is now a computationally mature procedure [2, 22, 23], growth maximization for ME models is a more difficult nonlinear program. Furthermore, ME models are inherently multiscale, as metabolic and protein expression processes operate at rates differing by 15 orders of magnitude. Thus, a major obstacle to the widespread adoption of ME models has been the complexity, speed and numerical reliability of simulation [9]. In this paper, we developed a quad-precision nonlinear programming (NLP)-based solution method exhibiting significant improvements in computational speed and numerical reliability. The quad-precision LP/NLP solver Quad MINOS [10] was used for this purpose. In addition, we developed quad-precision flux variability analysis [16] for ME models (varyME) using computationally efficient solver warm-start techniques [17]. We also showed that our solution methods were applicable to large-scale studies, including growth simulation on over 300 different media, and over 1400 gene deletion simulations.

With advances in ME solution methods enabling rapid prototyping, we anticipate the acceleration of ME model development for additional organisms, such as phototrophs [24]. Development may be further facilitated by possible extension of model reduction techniques [25] to ME models. Additionally, ME models will become more accessible to metabolic engineers and systems biologists, who have already stated the importance of proteome-accounting for improving strain design and optimization [26, 27].

To demonstrate possible application of our solution methodology in an engineering context, we analyzed growth-coupled succinate overproduction by *E. coli*. Unlike metabolic reconstructions that predict maximal product yield when the most metabolically efficient production pathway is used, the ME model predicted that simultaneous expression of multiple pathways led to the highest yields when proteome reallocation requirements were considered. These predictions were consistent with literature, where simultaneous expression of two succinate-producing pathways led to increased yield [21]. As shown here, ME models now enable genome-wide proteome-accounting for the simulation and design of microbes. Moving forward, it will be of interest to extend the theory and algorithms developed for metabolic models [28–30] to ME models.

## Conclusions

We developed an efficient methodology for solving nonlinear, multiscale models of metabolism and macromolecule expression (ME models). In particular, we showed that the growth rate maximization problem for ME models is a quasiconcave maximization problem in general and quasilinear for special cases as demonstrated here. In both cases, the global optimum is found efficiently using the projected Lagrangian method. Using the quad-precision LP/NLP solver Quad MINOS, we obtained up to 45 % speedup over conventional bisection. Currently, a phylogeny of in silico methods has emerged around metabolic reconstructions [31]. Our new insights into the quasiconvexity of nonlinear constraints in ME models and the reliability gained with quad-precision NLP solvers should help accelerate similar expansion of algorithms and applications using ME models in the near future.

## Methods

Serial computations were performed on 2.70 GHz Intel(R) Core(TM) i7-4800MQ processors with 8 GB of RAM and the Arch Linux operating system. Parallel computations were performed using resources of the National Energy Research Scientific Computing Center (NERSC).

### Quasiconvex and quasilinear optimization

For our reduced ME model, the dilution and growth rate-dependent protein-per-RNA constraints are gathered into the general form, *μAv*+*Bv*=*Bv*−*μCv*=0 (where *C*=−*A*). Here, we show that these constraints are quasilinear. First, recall that a quasilinear function is both quasiconvex and quasiconcave [12]. A quasiconvex function is a function whose *μ*-sublevel sets are all convex while a quasiconcave function has convex *μ*-superlevel sets. Accordingly, a quasilinear function has all convex *μ*-level sets. The *μ*-level set of constraint *i*∈*DIL* is

$$\begin{array}{*{20}l} L_{\mu} &= \left\{ v \left| \frac{{b_{i}^{T}} v}{{{c_{i}^{T}}} v}\right. = \mu \right\}\\ &= \left\{ v \left| {{b_{i}^{T}}} v - \mu {{c_{i}^{T}}} v\right. = 0 \right\}, \end{array} $$

which is a 0-level set of the sum of linear functions, i.e., a convex function (recall *μ* is fixed in the level set). Therefore, every *μ*-level set is convex; hence the dilution constraints are quasilinear.

ME models in [5, 6] have also implemented dilution constraints as inequalities: *Bv*−*μCv*≥0. In this case, the constraints are quasiconcave because the *μ*-superlevel sets are all convex:

$$\begin{array}{*{20}l} L^{+}_{\mu} &= \left\{ v \left| \frac{{{b_{i}^{T}}} v}{{{c_{i}^{T}}} v} \right.\ge \mu \right\}\\ &= \left\{ v \left| {{b_{i}^{T}}} v - \mu {{c_{i}^{T}}}\right. v \ge 0 \right\}. \end{array} $$

In this case, growth-rate maximization is a quasiconcave maximization (or quasiconvex minimization) problem whose global optimum is again found readily [12].

### Solution of LP and NLP problems

Quad MINOS 5.6 [10] was used to solve the LP and NLP problems in quad-precision. All Quad MINOS runs were performed with feasibility and optimality tolerances of 10^−15^. To determine orders of magnitude of ME fluxes (Table 5), we used feasibility and optimality tolerances of 10^−20^. To interface the Fortran-based Quad MINOS shared library and custom Fortran source code to the cobrapy [32] Python package, we used the f2py [33] function in Numpy 1.8.1. Solution of ME models in double-precision was performed using IBM ILOG CPLEX 12.6.0. Bisection was performed with the initial interval [*a*,*b*]=[0,2].

### Biological conditions for ME simulations

ME simulations were performed with an uptake rate of –1000 mmol/gDW/h for substrates available in the medium (carbon, nitrogen, phosphorus, sulfur sources) and for oxygen uptake. Unlike metabolic models that require physiologically determined uptake rate bounds, ME models predict a limit to maximum uptake. This maximum uptake occurs at the max growth rate where additional RNA and protein machinery cannot be produced to meet growth demands [5].

### Orders of magnitude spanned by ME fluxes

To estimate the orders of magnitude spanned by ME fluxes, we first found the smallest transcription flux for genes determined to be essential experimentally and by the ME simulation. Because multiple transcription units (TUs) can encode a gene, and multiple genes can be encoded by a TU, we found the smallest transcription flux greater than the solver feasibility tolerance (10^−20^) encoding an essential gene. On glucose minimal medium, this flux was 9.35×10^−14^ mmol/gDW/h. Note that the smallest translation flux was 9.13×10^−13^ mmol/gDW/h. The maximum flux was 45.4 mmol/gDW/h, for H_2_O exchange. Thus, on glucose minimal medium, fluxes spanned nearly 15 (14.7) orders of magnitude.

### Reduced ME model of *E. coli*

The reduced ME model of *E. coli* was developed separately [34] and was used to facilitate rapid algorithm testing throughout this study. The stoichiometric matrix consists of 8757 rows and 16,126 columns. Compared to the ME model of *T. maritima* (18,209 rows × 17,535 columns), the reduced ME model of *E. coli* has a similar number of reactions but considerably fewer constraints. The reduced ME model has a number of simplifications compared to the full ME models of *E. coli*: iOL1650 [5] (68,726 rows × 76,413 columns) and iJL1678 [6] (70,751 rows × 79,871 columns). The model includes the majority of metabolic and expression machinery processes, as well as core and niche proteome genes needed for growth in diverse environments [20]. The full ME model by O’Brien et al. [5] included growth rate-dependent cell wall dilution, which added a nonlinear constraint that was more complicated than the bilinear constraints arising from macromolecule dilution. This constraint is excluded in the reduced model. Also excluded are explicit degradation reactions for mRNA.

## Additional files

Additional file 1
**Figure S1.**(PDF 5 kb)

Additional file 2
**Tables S1–S2.**(XLSX 49 kb)
