# Supplementary figures and images for: solveME: fast and reliable solution of nonlinear ME models
Source: BMC Bioinformatics. 2016 Sep 22;17:391. doi: 10.1186/s12859-016-1240-1 (PMC5034503; doi:10.1186/s12859-016-1240-1)

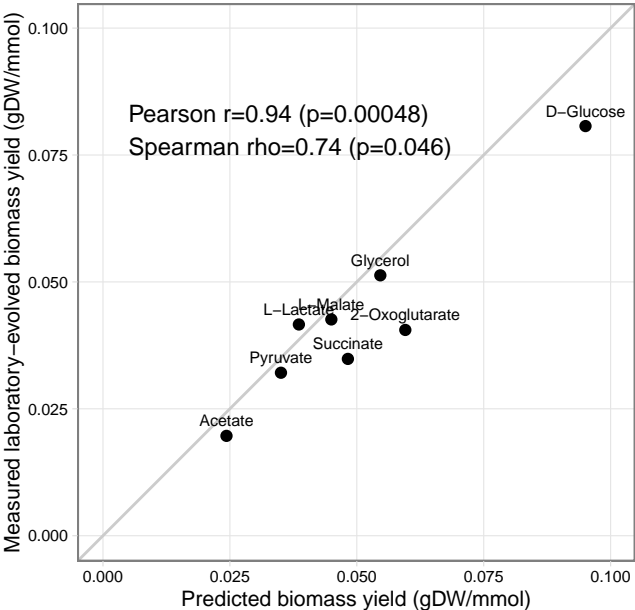

Supplement: Additional file 1 — Figure S1.(PDF 5 kb) [file 12859_2016_1240_MOESM1_ESM.pdf]
